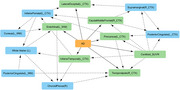# Identifying brain regions to characterize AD using PET tracer uptake: A Bayesian approach

**DOI:** 10.1002/alz70856_100736

**Published:** 2025-12-25

**Authors:** Geetha Chilla

**Affiliations:** ^1^ Bioinformatics Institute, A*STAR, Singapore, Singapore, Singapore

## Abstract

**Background:**

Amyloid plaque deposition in Alzheimer's Disease is commonly assessed using PET imaging, with the Centiloid scale serving as the standard measure for quantifying global amyloid burden. However, in addition to the Centiloid, tracer uptake in brain subregions could also provide information about the disease and its extent. In this work, we aimed to identify such brain regions whose tracer uptake information could serve as markers for AD diagnosis.

**Method:**

The study uses PET imaging data (tracer: Pittsburgh Compound B) from the OASIS‐3 dataset, consisting of 44 cognitively normal controls and 35 mild‐moderate AD patients. The images were co‐registered to Freesurfer parcellated MRI scans and the standard uptake value ratio (SUVR) was calculated in subregions ROI, and were corrected for partial volume effects. Using Bayesian methods, a minimal but highly predictive subset of features are first identified, from which key variables associated with disease status are extracted using Markov blanket.

**Result:**

SUVRs of 23 brain regions (in addition to Centiloid) were identified as minimal feature marker subset, that are highly predictive of AD diagnosis. 6 among these 23 subregions SUVRs are directly linked to disease status in addition to global amyloid deposition measure (Centiloid). The predictive features that are strongly associated with AD include PIB uptake in regions such as Precuneus, Inferior temporal, lateral occipital and posterior cingulate regions of left cortex, supramarginal and temporal regions of right cortex and right choroid plexus.

**Conclusion:**

In this work, regional standardized uptake value ratios (SUVR) from PIB‐PET scans are analyzed to examine key brain regions linked to AD diagnosis. Tracer uptake in 13 of these regions and global amyloid deposition are identified to be highly linked to disease status, that could serve as markers and inform about the likelihood of AD in patients. Future studies could explore tracer uptake in these regions in larger cohorts to confirm their utility in AD diagnosis and treatment.

Data was provided by OASIS‐3 (LaMontagne et al., 2019) and all analyses were carried out on AD Workbench (2020), Alzheimers Disease Data Initiative (https://www.alzheimersdata.org/). This work was funded by the AD Data Initiative.